# Calcium-Sensing Receptor in Breast Physiology and Cancer

**DOI:** 10.3389/fphys.2016.00440

**Published:** 2016-09-30

**Authors:** Wonnam Kim, John J. Wysolmerski

**Affiliations:** Section of Endocrinology and Metabolism, Department of Internal Medicine, Yale University School of MedicineNew Haven, CT, USA

**Keywords:** calcium-sensing receptor, breast cancer, parathyroid hormone-related protein, mammary gland, lactation

## Abstract

The calcium-sensing receptor (CaSR) is expressed in normal breast epithelial cells and in breast cancer cells. During lactation, activation of the CaSR in mammary epithelial cells increases calcium transport into milk and inhibits parathyroid hormone-related protein (PTHrP) secretion into milk and into the circulation. The ability to sense changes in extracellular calcium allows the lactating breast to actively participate in the regulation of systemic calcium and bone metabolism, and to coordinate calcium usage with calcium availability during milk production. Interestingly, as compared to normal breast cells, in breast cancer cells, the regulation of PTHrP secretion by the CaSR becomes rewired due to a switch in its G-protein usage such that activation of the CaSR increases instead of decreases PTHrP production. In normal cells the CaSR couples to Gα_i_ to inhibit cAMP and PTHrP production, whereas in breast cancer cells, it couples to Gα_s_ to stimulate cAMP and PTHrP production. Activation of the CaSR on breast cancer cells regulates breast cancer cell proliferation, death and migration, in part, by stimulating PTHrP production. In this article, we discuss the biology of the CaSR in the normal breast and in breast cancer, and review recent findings suggesting that the CaSR activates a nuclear pathway of PTHrP action that stimulates cellular proliferation and inhibits cell death, helping cancer cells adapt to elevated extracellular calcium levels. Understanding the diverse actions mediated by the CaSR may help us better understand lactation physiology, breast cancer progression and osteolytic bone metastases.

## Introduction

The extracellular calcium-sensing receptor (CaSR) is a G-protein-coupled receptor (GPCR) that was first identified because of its ability to regulate parathyroid hormone (PTH) secretion in response to changes in extracellular free calcium (Ca^2+^) (Brown et al., 1993; Brown, 2013). It belongs to class C of the GPCR superfamily, which also includes metabotropic glutamate receptors (mGluRs), GABA B receptors, taste receptors and pheromone receptors (Pi et al., 2005; Brauner-Osborne et al., 2007). The CaSR has been shown to bind and signal in response to extracellular Ca^2+^ concentrations within the physiologic range (Brown and MacLeod, 2001). It can also be activated by a variety of other cations and cationic compounds such as gadolinium, amino acids, spermine and certain antibiotics (Brown and MacLeod, 2001; Ward et al., 2002). The receptor appears to act as a homodimer, or perhaps a tetramer, and has also been shown to heterodimerize to form hybrid, signaling complexes with other class C GPCRs (Bai et al., 1998, 1999; Gama et al., 2001; Chang et al., 2007). The CaSR complex is sensitive to allosteric modulation that can alter its sensitivity to direct agonists such as extracellular Ca^2+^, an aspect of its biology that has been exploited to develop therapeutic agents such as Cinacalcet (Brown and MacLeod, 2001; Filopanti et al., 2013).

The CaSR is highly expressed by parathyroid glands, bone cells and the kidney where it acts to regulate PTH secretion, bone turnover and renal calcium handling in order to coordinate systemic calcium metabolism and to maintain a stable Ca^2+^ concentration in the extracellular fluid. In addition to its role as a master regulator of calcium metabolism, a large body of evidence also supports its involvement in the regulation of diverse processes, such as cellular proliferation, differentiation, apoptosis, hormone secretion, gene expression and ion transport in many different organs (Brown and MacLeod, 2001; Hofer and Brown, 2003; Brennan and Conigrave, 2009; Breitwieser, 2012; Chakravarti et al., 2012; Brennan et al., 2013). Interestingly, at sites other than the parathyroid gland, the CaSR often regulates the secretion of parathyroid hormone-related protein (PTHrP) (Wysolmerski, 2012), a locally produced cytokine growth factor that is evolutionarily related to PTH and uses the same Type 1 PTH/PTHrP receptor (PTH1R). In this review we will focus on the functions of the CaSR in mammary gland biology, its regulation of mammary gland PTHrP production and its contribution to the development and progression of breast cancer.

## The CaSR in the normal breast

### CaSR expression in normal mammary epithelial cells

The first documentation of CaSR expression in normal breast tissue was reported by Cheng et al. (1998). They demonstrated both CaSR mRNA and protein expression in ductal epithelial cells in the human breast. Subsequent studies in mice confirmed these observations and showed that CaSR mRNA levels in the mouse mammary gland are low during ductal development and early to mid-pregnancy but subsequently increase to a peak during lactation (VanHouten et al., 2004). It is not entirely clear whether these changes represent alterations in the proportion of the gland represented by epithelial cells at these different time points, an actual increase in the expression of CaSR mRNA in individual epithelial cells, or both. Either way, the expression of the CaSR is rapidly reduced after weaning of the pups and the cessation of lactation. Cheng and colleagues demonstrated CaSR staining on epithelial cells in human breast samples (Cheng et al., 1998). Likewise, in mice, CaSR immunofluorescence staining demonstrated that, during lactation, the receptor is expressed primarily on the basolateral surface of ductal and alveolar cells as well as within the intracellular compartment of these same cells (VanHouten et al., 2004, 2007). Although these studies do not rule out low levels of receptor in stromal cells, it appears that the CaSR is located predominantly on epithelial cells within the breast.

### CaSR function in the normal mammary gland

Our laboratory has disrupted the CaSR selectively in mammary epithelial cells, using both MMTV-Cre and BLG-Cre mice. In MMTV-Cre;CaSR^lox/lox^ mice, the CaSR gene is disrupted in mammary epithelial cells soon after weaning. This caused no apparent abnormalities in ductal development during puberty or alveolar development during pregnancy (Kim et al., 2016). In contrast, in BLG-Cre;CaSR^lox/lox^ mice, the CaSR gene is disrupted in mammary epithelial cells at the transition from pregnancy to lactation. While this did not affect secretory differentiation of the epithelial cells, it did have consequences for calcium transport, PTHrP production and systemic calcium metabolism during lactation, confirming previous pharmacologic and genetic studies (VanHouten et al., 2004, 2007; Ardeshirpour et al., 2006; Mamillapalli et al., 2013). These studies suggest that, despite its effects on proliferation and apoptosis in breast cancer cells (see below), the CaSR does not have a dominant role in regulating morphological development or differentiation in the normal mammary gland. Therefore, we will limit further discussion to the role of the CaSR in mammary gland physiology during lactation.

#### Regulation of PTHrP production

Parathyroid hormone-related protein (PTHrP, gene symbol *PTHLH*) was discovered as the cause of humoral hypercalcemia of malignancy (HHM) a common paraneoplastic syndrome (Wysolmerski, 2012). It has important functions during mammary gland development and also participates in the regulation of systemic calcium metabolism during lactation. PTHrP is produced by mammary epithelial cells and is secreted both into milk and into the systemic circulation. Circulating PTHrP activates bone resorption during lactation in order to liberate maternal skeletal calcium stores that are used by the mammary gland for milk production (Wysolmerski, 2012). PTHrP in milk modulates neonatal calcium accrual through unknown mechanisms (Mamillapalli et al., 2013). The expression of *Pthlh* mRNA by the lactating murine mammary gland and the secretion of PTHrP into the maternal circulation and into milk are regulated by the CaSR so that PTHrP production by mammary epithelial cells is ultimately responsive to the availability of calcium for milk synthesis (VanHouten et al., 2004; Ardeshirpour et al., 2006; VanHouten and Wysolmerski, 2007; Mamillapalli et al., 2013; VanHouten and Wysolmerski, 2013).

As noted in the Introduction, the CaSR has been shown to regulate PTHrP production by several cell types, such as astrocytes, ovarian epithelial cells, cytotrophoblasts, hepatocytes, osteoblasts, prostate cancer cells, CaSR transfected HEK293 cells, and breast cancer cells (Brown and MacLeod, 2001; Chattopadhyay, 2006; Reyes-Ibarra et al., 2007; Wysolmerski, 2012; Organista-Juarez et al., 2013). In most cell types studied, activation of the CaSR stimulates PTHrP production. However, in normal mammary epithelial cells, activation of the CaSR suppresses PTHrP production (Sanders et al., 2000; VanHouten et al., 2004). Our laboratory, as well as others, has demonstrated this effect in the intact mammary gland as well as in isolated mammary epithelial cells in cell culture using both genetic and pharmacologic approaches. For example, reducing dietary calcium intake in lactating wild-type mice increases *Pthlh* gene transcription and PTHrP secretion into milk. Increasing dietary calcium intake in lactating WT, PTH^−/−^, 1α(OH)ase^+/−^ or 1α(OH)ase^−/−^ mice did the opposite and decreased PTHrP concentrations, demonstrating a consistent inverse relationship between circulating calcium concentrations and mammary PTHrP levels in lactating mice (Cao et al., 2009; Ji et al., 2011; VanHouten and Wysolmerski, 2013). Infusion of the calcimimetic compound NPS-R467, an allosteric activator of the CaSR, in lactating mice fed a low calcium diet recovered PTHrP production to levels observed in control mice fed a normal calcium diet, demonstrating that these dietary manipulations regulate mammary PTHrP production through the CaSR (VanHouten et al., 2004). We have also observed similar findings in genetic models of CaSR deficiency. Homozygous disruption of the CaSR gene results in neonatal death, but the mammary glands of CaSR^+/−^ mice produce more PTHrP during lactation than WT mice (Ardeshirpour et al., 2006). To circumvent the neonatal death of CaSR^−/−^ mice, we used the BLG-Cre transgene to disrupt the floxed *Casr* gene during late pregnancy and lactation (Mamillapalli et al., 2013). Loss of the CaSR on mammary epithelial cells resulted in increased *Pthlh* mRNA expression, increased milk PTHrP levels and increased secretion of PTHrP into the maternal circulation. All together, these studies support the conclusion that activation of the CaSR on mammary epithelial cells suppresses PTHrP production and secretion into the circulation and milk (VanHouten and Wysolmerski, 2013). As discussed below, this defines a negative feedback loop between calcium delivery to the lactating mammary gland and PTHrP production by mammary epithelial cells.

The CaSR binds Ca^2+^ mainly through the large extracellular domain (ECD) and acts as a homodimer or heterodimer (Bai et al., 1998, 1999; Chakravarti et al., 2012). Upon ligand binding, the CaSR undergoes a conformational change, which promotes GDP dissociation from a Gα subunit of a heterotrimeric G-protein complex, causing it to dissociate from the Gβγ subunits. As with many GPCRs, the CaSR interacts with multiple Gα sub-types, and the downstream signaling pathways are highly divergent, and depend on the cellular context (Chakravarti et al., 2012). The CaSR has been shown to stimulate the PLC/PKC pathway downstream of Gα_q_ and to decrease cAMP downstream of Gα_i_ (Chakravarti et al., 2012). Both Gα_q_ and Gα_i_, as well as interaction with the scaffolding proteins filamin-A and caveolin-1, are thought to be involved in the inhibition of PTH secretion by the CaSR in parathyroid chief cells (Chakravarti et al., 2012). In several cell types, the CaSR has been suggested to regulate PTHrP secretion by modulating MAPK signaling (Tfelt-Hansen et al., 2003). However, in normal mammary epithelial cells, regulation of cAMP/PKA pathways appears to be more important for PTHrP production than changes in the MAPK/ERK pathway (Mamillapalli et al., 2008). Our laboratory has demonstrated that, in normal breast cells, the CaSR couples with Gα_i_ to decrease cAMP production by adenylylcyclase, and subsequently PKA activation, without affecting phosphodiesterase activity (Mamillapalli et al., 2008). Lower cAMP production results in the inhibition of *Pthlh* gene expression and PTHrP secretion, an effect that can be mimicked by inhibition of PKA, or reversed with forskolin or dibutyryl-cAMP (Mamillapalli et al., 2008).

#### Regulation of calcium transport into milk

Milk production requires the transport of large amounts of calcium from the maternal circulation into milk. There is little paracellular transport in the lactating mammary gland and calcium must pass through the epithelial cells to enter the lumen (VanHouten and Wysolmerski, 2007). The plasma membrane calcium-ATPase 2 (PMCA2), a P-type, ion transport ATPase pump is expressed on the apical surface of mammary epithelial cells and transports 60–70% of milk calcium from the epithelial cells across the apical membrane and into the acinar lumen (Reinhardt et al., 2004; VanHouten et al., 2007). The remainder of the calcium in milk appears to be pumped into the endoplasmic reticulum or secretory granules and to be secreted bound to caseins via the exocytosis of casein-containing granules. The CaSR regulates transcellular calcium transport by altering the activity of PMCA2 in mammary epithelial cells (VanHouten et al., 2007; VanHouten and Wysolmerski, 2007). In this instance, delivery of calcium to the mammary gland activates the CaSR, which, in turn, stimulates PMCA2 activity and increases calcium transport into milk. The intracellular signal transduction pathways linking the CaSR to PMCA2 activation are unknown and require further study (VanHouten and Wysolmerski, 2013). It has been suggested that calcium enters mammary epithelial cells through interactions between another calcium pump, SPCA2, and the store-operated calcium channel, ORAI1 (Cross et al., 2013; Ross et al., 2013). At this juncture, it is also unknown whether CaSR signaling regulates this complex to increase calcium entry into the epithelial cells. However, the available data clearly demonstrate that the CaSR regulates the amount of calcium transported into milk in a positive feedback loop that is responsive to the availability of maternal calcium supplies (VanHouten and Wysolmerski, 2013).

#### Systemic aspects of the CaSR-PTHrP and CaSR-PMCA2 feedback loops during lactation

**Figure 1:**
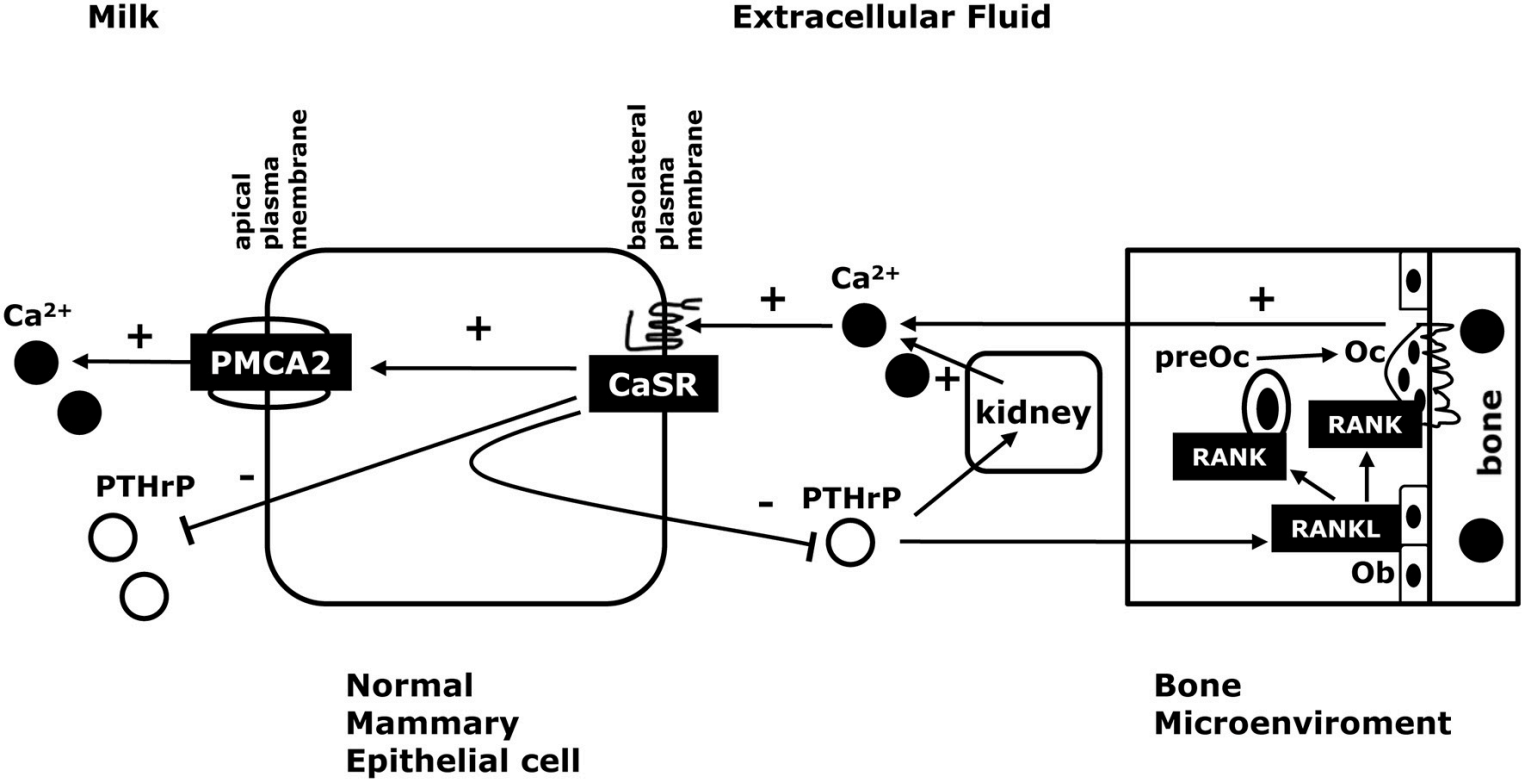
**The CaSR in normal lactation**. Activation of the CaSR in the lactating breast promotes trans-epithelial calcium transport into milk by increasing the activity of the calcium pump, PMCA2. In addition, activation of the CaSR suppresses PTHrP release into milk and into the systemic circulation. Circulating PTHrP acts on osteoblasts (Ob) to increase osteoclast (Oc) development and activity through the RANK/RANKL pathway. This, in turn, stimulates bone resorption and liberates skeletal calcium stores. PTHrP also acts on the kidney to stimulate calcium reabsorption. If serum calcium is decreased, the CaSR decreases calcium transport into milk to decrease calcium utilization and increases PTHrP secretion into the maternal circulation to increase the supply of calcium from skeletal stores. In this way, the CaSR helps to maintain a steady supply of calcium for milk production and protects the mother from hypocalcemia if dietary calcium become limiting.

Current data suggest that the CaSR and PTHrP are involved in a negative feedback loop that regulates the supply of calcium to the mammary gland to support milk production (see Figure 1; VanHouten, 2005; VanHouten and Wysolmerski, 2007, 2013). The effects of the CaSR on PTHrP production in the lactating mammary gland mimic the relationship between Ca^2+^, the CaSR and PTH production by the parathyroid glands. In essence, it can be argued that the lactating breast serves as an accessory parathyroid gland that secretes PTHrP into the maternal circulation in a calcium-sensitive manner in order to regulate osteoclastic bone resorption and the release of stored calcium from the maternal skeleton into the circulation to be available for uptake by the mammary gland and secretion into milk. In addition, the CaSR stimulates calcium transport into milk, allowing the lactating mammary gland to transport more calcium when it is available and to decrease its calcium usage when the mother's supply of calcium becomes limiting. Ultimately, we believe that this combined control of calcium mobilization and calcium usage allows the CaSR to fine tune maternal calcium metabolism to ensure a steady supply of calcium for milk production but to protect against maternal hypocalcemia. If calcium is readily available in the diet, more calcium is transported into milk but less PTHrP is produced and less calcium is liberated from skeletal stores. However, when dietary calcium is less abundant, the mammary gland reduces milk calcium content by slowing calcium transport and more PTHrP is produced, which increases bone resorption and liberates further calcium from skeletal stores. The importance of changes in milk PTHrP are less clear. However, recent studies have demonstrated that milk PTHrP content correlates inversely with milk calcium content and neonatal calcium accrual (Mamillapalli et al., 2013). We hypothesize that milk PTHrP may serve as a metabolic message that entrains maternal and neonatal bone and calcium metabolism. If less calcium is available to the mother, she reduces the calcium content of milk but increases milk PTHrP secretion, which, in turn, reduces neonatal calcium usage. If calcium is abundant, then milk calcium increases and milk PTHrP decreases, allowing increased neonatal calcium accrual. While the initial data support this model, much more work is needed to fully understand how milk calcium and PTHrP content regulate neonatal calcium and bone metabolism.

## The CaSR in breast cancer

Breast cancer is a common malignancy in women that has been estimated to afflict one in eight women in the United States. The American Cancer Society estimates that, in 2016, 249,260 women in the United States will be diagnosed with breast cancer and approximately 40,890 will die from this disease (https://cancerstatisticscenter.cancer.org/). A particularly difficult problem in breast cancer is the frequent occurrence of bone metastases, which affect up to 65% to 80% of women with advanced disease (Weilbaecher et al., 2011). The ability of breast cancer cells to grow in bone has been shown to be related to their ability to recruit osteoclasts to resorb bone around the tumor deposit. This causes the release of growth factors from the bone matrix into the microenvironment around tumor cells, which increase their proliferation in response. The increased numbers of tumor cells then stimulate further osteolysis, which accommodates the growth of the tumor within the bone and releases even more growth factors to further feed tumor growth (Chen et al., 2010; Patel et al., 2011; Weilbaecher et al., 2011). This positive-feedback relationship between breast cancer cells and bone is referred to as a vicious cycle of osteolysis. This vicious cycle involves complex interactions between breast cancer cells, immune cells, osteoblasts and osteoclasts, mediated by a variety of biochemical signaling pathways and non-coding RNAs (Keklikoglou et al., 2012; Taipaleenmaki et al., 2015; Martin, 2016). Investigators have implicated both the CaSR and PTHrP as among the molecules contributing to the pathophysiology of osteolytic bone metastases (Yin et al., 1999; Mihai et al., 2006). In this Section, we will review current evidence suggesting that the CaSR regulates PTHrP production and tumor growth in breast cancer as well as recent findings suggesting that a CaSR-nuclear PTHrP signaling pathway may contribute to the proliferation and survival of breast cancer cells in high-calcium environments, such as bone.

### CaSR expression in breast cancer

Several studies have shown that the CaSR is expressed in breast carcinomas from patients and in breast cancer cell lines. CaSR expression was first demonstrated on an mRNA and protein level in breast tissue samples from 6 different women: 2 samples of normal breast tissue, 2 samples of fibrocystic disease, and 2 samples of ductal carcinomas (Cheng et al., 1998). Subsequent reports documented CaSR expression in standard human breast cancer cell lines, such as MCF-7 and MDA-MB-231 cells (Sanders et al., 2000). Some studies have shown that fully transformed MCF-7 and MDA-MB-231 breast cancer cell lines had higher CaSR levels than the nonmalignant breast cell lines, Hs578Bst and MCF-10A cells (Huang et al., 2009; VanHouten and Wysolmerski, 2013). In addition, we examined data from a study of rat mammary tumors induced by the chemical carcinogen N-Nitroso-N-methylurea (NMU) (Chan et al., 2005) and found that *Casr* mRNA levels were significantly higher in tumors as compared to normal mammary tissue (Kim et al., 2016). In contrast, recent studies from the Yang laboratory have suggested that human breast cancers may express lower levels of CaSR than normal breast tissue (Li et al., 2014a,b). In addition, they found that the “AG” or “GG” genotype at SNP rs17251221 was associated with reduced CaSR protein levels in 15 tumors and an increase in the risk of breast cancer in a case-control study of Chinese patients with (*n* = 217) or without (*n* = 231) breast cancer (Li et al., 2014a). Interestingly this same intronic polymorphism has been associated with prostate and ovarian cancer risk as well (Jorde et al., 2013; Jeong et al., 2016). A different study in 3663 cases and 4687 controls of women of African American descent did not replicate these associations but did find that another CaSR SNP (rs112594756) was associated with estrogen receptor status in breast tumors (Yao et al., 2016). A study that looked at CaSR immunohistochemistry in histological sections of primary tumors from 65 patients with metastatic breast cancer showed that the levels of CaSR expression varied among the different tumors and that these differences correlated with the biological behavior of the tumors. Primary tumors with high levels of CaSR expression were associated with bone metastases while those with lower levels of CaSR expression were not (Mihai et al., 2006). Finally, we examined CaSR protein expression using the semi-automated immunofluorescence AQUA platform in a tissue microarray consisting of 652 breast tumors with a median clinical follow up of 8.9 years (YTMA49) (Camp et al., 2002; VanHouten et al., 2010). In YTMA49, CaSR levels correlated inversely with pathological progesterone receptor positive status and positively with node-positive status. CaSR levels above the median value were associated with significantly shorter survival than those below the median, although, when considered as a continuous variable, CaSR levels did not significantly predict survival (Kim et al., 2016).

Other than to say that the CaSR is expressed in breast cancers and that there may be associations between CaSR expression and different aspects of breast cancer behavior, it is difficult to synthesize the above data into a coherent picture. One possibility is that decreased expression of the CaSR may be associated with an increased risk of developing breast cancer but, once breast cancer is established, tumor levels of CaSR may alter behaviors such as the ability to grow in bone. However, the results from the above case-control studies are based on relatively small numbers and larger studies will be required to determine whether increased or decreased levels of CaSR expression alter breast cancer risk. Furthermore, we did not find that ablation of the *Casr* gene in MMTV-PyMT transgenic mice increased the incidence of mammary tumors (see below) (Kim et al., 2016). It should also be noted that SNPs in the *CASR* gene locus have not been associated with breast cancer risk in GWAS studies (Fachal and Dunning, 2015). Very little is known about the regulation of CaSR expression in breast cancer cells and, therefore, we do not yet understand the mechanisms determining the level of CaSR expression in individual cancers. One study showed that BRCA1 regulates the expression of CaSR in MCF-7 and MDA-MB-231 cells but it is not known whether breast tumors in patients with BRCA1 mutations have different levels of CaSR expression than non-BRCA1-associated tumors (Promkan et al., 2011). Clearly, given the suggestion that CaSR levels might influence the likelihood of breast cancer risk, the estrogen-receptor status of tumors and/or the growth of bone metastasis, these issues need to be explored in larger studies with sufficient power to examine the different genetic sub-types of breast cancer individually.

### Function of the CaSR in breast cancer

#### Proliferation and cell death

Studies examining the effects of CaSR signaling on proliferation and cell death in breast cancer cells have reported contradictory results. A majority of studies have focused on cell proliferation and different labs have reported opposite results sometimes using the same cell lines. In one study, Ca^2+^ concentrations between 1.4 and 5.0 mM stimulated the proliferation of MCF-7 cells by 25 ± 3% as assessed by measuring cell accumulation (El Hiani et al., 2009a). These authors described a pathway by which stimulation of the CaSR activated membrane metalloproteinases (MMPs), which led to shedding of membrane-bound EGF-family growth factors, stimulation of the EGFR, ERK1/2 phosphorylation, upregulation of the transient receptor potential channel 1 (TRPC1) and the stimulation of cell proliferation (El Hiani et al., 2009b). It has been well documented that GPCRs can transactivate EGFRs both in ligand-dependent and ligand-independent signaling pathways (Bhola and Grandis, 2008), and the CaSR has been reported to activate the EGFR in this manner in leydig cancer cells, fibroblasts, and prostate cancer cells suggesting that this could be a commonly utilized signaling mechanism (Yano et al., 2004; Tfelt-Hansen et al., 2005; Tomlins et al., 2005). Activation of the CaSR in MCF-7 and MDA-MB-231 breast cancer cells has also been shown to increase the production of phosphocholine and the expression of choline kinase through activation of Gα_12_ and Rho (Huang et al., 2009). Furthermore, overexpression of choline kinase in MCF-7 breast cancer cell has been shown to increase their invasiveness and drug resistance (Shah et al., 2010). The regulation of phosphocholine synthesis seems to be operative in human tumors *in vivo* as Baio and colleagues have used MRI spectroscopy in 23 patients to demonstrate a positive correlation between the pre-operative choline content of breast cancers and the staining intensity of CaSR immunohistochemistry on pathological specimens after surgery (Baio et al., 2015). Elevated levels of phosphocholine and total choline-containing compounds have been observed in almost every cancer type studied and may be associated with tumor progression (Glunde et al., 2011). In the aggregate, these data support that notion that the CaSR promotes increased proliferation of breast cancer cells and suggest two interesting signaling pathways that may mediate these effects.

In contrast to the above studies, other reports have suggested that CaSR signaling has no effect, or reduces proliferation and decreases the malignant behavior of breast cancer cells (Liu et al., 2009; Promkan et al., 2011). Sanders et al. examined responses to varying concentrations of Ca^2+^ between 0.5 and 10 mM and found that changing extracellular calcium had no effect on cell proliferation in MCF-7 or MDA-MB-231 cells as assessed by cell accumulation (Sanders et al., 2000). In a different study, Liu and colleagues found that exposing MCF-7 cells to physiological concentrations of Ca^2+^ (1.4 mM) reduced proliferation (as assessed by changes in cell numbers) as compared to low concentrations of Ca^2+^ (0.175–0.2 mM) (Liu et al., 2009). These investigators have also reported that activation of the CaSR increased the sensitivity of MCF-7 and MDA-MB-231 breast cancer cells to cell death in response to paclitaxel (Liu et al., 2009; Promkan et al., 2011). Similar findings have been reported in colon cancer, where it has been proposed that activation of the CaSR inhibits tumor progression and enhances chemotherapeutic responses (Rogers et al., 2012). The growth inhibitory effects of the CaSR in breast cancer cells have been reported to be mediated by the downregulation of the expression of survivin, a well described anti-apoptotic factor in breast and other cancers (Liu et al., 2009; Promkan et al., 2011). Interestingly, in these studies, wild-type BRCA1 was shown to inhibit survivin expression in a CaSR-dependent manner (Promkan et al., 2011). Data from these several studies suggest that the CaSR inhibits proliferation and promotes cell death in breast cancer cell lines, implying that it may inhibit breast cancer development and/or progression.

It is unclear how to interpret the contradictory findings suggesting that the CaSR both opposes and promotes proliferation and/or cell death, especially when groups reporting opposite findings have used the same cell lines. In essence, one can find data showing that high Ca^2+^ stimulates, inhibits or has no effect on the proliferation of MCF-7 or MDA-MB-231 cells. The reported differences might be explained by differences in the Ca^2+^ concentration used by the different groups (El Hiani et al., 2009a,b; Liu et al., 2009; Promkan et al., 2011) or they may be the consequence of complex patterns of downstream signaling that might be very context dependent (Mamillapalli et al., 2008; Promkan et al., 2011; Breitwieser, 2012; Chakravarti et al., 2012).

#### Regulation of cell migration

The development of distant metastases is perhaps the most significant cause of cancer mortality. Metastasis is a multi-step process, which requires cells to migrate in order for them to successfully invade surrounding tissues, to disseminate widely and to colonize other tissue and form metastases. Several studies have suggested that the CaSR can promote cell migration and therefore may promote the development of metastases. Using the Boyden Chamber and Scratch Wound migration assays, Saidak et al. showed that stimulation of the CaSR with extracellular calcium concentrations between 1.8 and 5.0 mM stimulated the migration of MCF7, T47D and MDA-MB-231 breast cancer cells (Saidak et al., 2009). Interestingly, of these 3 cell lines, the highly bone metastatic MDA-MB-231 cells showed the most vigorous cell migration response. Knocking down CaSR expression inhibited cell migration in response to Ca^2+^ in all three, cell lines demonstrating that the CaSR mediated the migratory effect of extracellular calcium (Saidak et al., 2009). Activation of the CaSR appeared to enhance migration in these cells by activating the ERK1/2, MAPK and phospholipase Cβ (PLCβ) pathways. Activation of the CaSR has also been shown to induce the secretion of a series of pro-angiogenic cytokines by MDA-MB-231 cells, at least in part, through activation of EGFR signaling (Hernandez-Bedolla et al., 2015). In this instance the CaSR appears to indirectly regulate the migration and morphogenesis of endothelial cells and may contribute to tumor neovascularization. The CaSR has also been shown to affect migration in other cell types. In medullary thyroid carcinoma cells, the CaSR interacts with β1 integrin, modulating cellular adhesion and inducing migration, through a PLC/intracellular calcium-mediated pathway (Tharmalingam et al., 2011). In keratinocytes, the CaSR physically interacts with Trio, Rho, and filamin A to form a signaling complex that regulates E-cadherin-mediated cell-cell adhesion and differentiation (Tu and You, 2014). Finally, the CaSR also has been shown to modulate E-cadherin's membrane localization and binding with β-catenin in colon carcinoma cells (Wang et al., 2010). In the aggregate, these findings suggest that the CaSR may regulate cell migration in breast cancer cells, however, much more work must be done in this area to determine how important these observations *in vitro* are to the behavior of breast cancers *in vivo*.

#### Regulation of PTHrP production

Activation of the CaSR in normal mammary epithelial cells reduces PTHrP mRNA expression and PTHrP secretion (VanHouten et al., 2004; Ardeshirpour et al., 2006; Mamillapalli et al., 2008; 2013). However, just the opposite has been observed for breast cancer cells. In immortalized or malignant breast cells, such as Comma D, MCF-7, BT474 and MDA-MB-231 cells, activation of the CaSR with Ca^2+^, spermine, aminoglycoside antibiotics, or allosteric (type-II) calcimimetics has been shown to stimulate PTHrP secretion (Sanders et al., 2000; Mamillapalli et al., 2008; Kim et al., 2016). Interestingly, activation of the CaSR can act synergistically with TGFβ to increase PTHrP secretion in hepatocytes as well as MCF-7 and MDA-MB-231 breast cancer cells, a characteristic that may contribute to the pathogenesis of bone metastasis (Sanders et al., 2000; Organista-Juarez et al., 2013). Our laboratory has been interested in understanding the mechanisms by which malignant transformation of mammary epithelial cells causes CaSR activation to switch from inhibiting PTHrP production to stimulating PTHrP production. Initial studies demonstrated that divergent cAMP production, but not alterations in MAPK or PLC signaling correlated with the changes in PTHrP production (Mamillapalli et al., 2008). Activation of the CaSR decreases cAMP levels in normal breast cells, but increases cAMP response in breast cancer cells (Mamillapalli et al., 2008; Kim et al., 2016). We found that in normal breast cells, the CaSR couples to Gα_i_ and inhibits adenylyl cyclase while, in breast cancer cells, the CaSR couples to Gα_s_ and stimulates cAMP production. The *Pthlh* gene is known to be regulated by a cAMP-response element located within exon 4 of the human gene, and the effects of CaSR activation on PTHrP production in these different cell types could be mimicked by manipulating cAMP levels independently from CaSR activation (Chilco et al., 1998; Mamillapalli et al., 2008). Therefore, it appears that during the transformation of normal breast cells into malignant breast cancer cells, the CaSR switches its G-protein preference, which, in turn, leads to completely opposite effects on PTHrP production. Ongoing studies are attempting to define the molecular mechanisms that underlie the G-protein switching that occurs in response to malignant transformation.

#### A CaSR-nuclear PTHrP pathway regulates cell proliferation and survival

Given that the CaSR upregulates PTHrP and given that PTHrP has been shown to regulate breast cancer progression, we were interested in determining whether PTHrP might mediate some of the effects of the CaSR on breast cancer cells *in vitro* and *in vivo*. In order to examine this question, we first asked whether there was an association between CaSR and PTHrP levels in breast cancers. In NMU-induced mammary tumors in rats, there was a clear positive correlation between *Casr* and *Pthlh* mRNA levels. This was also true in human breast cancers at both an mRNA and protein level; *CASR* gene expression correlated directly with *PTHLH* gene expression in a gene array study of 204 human breast tumors (Mu et al., 2012; Kim et al., 2016) and CaSR and PTHrP protein expression showed a positive correlation in the YTMA49 tissue array of 652 breast tumors discussed previously (Camp et al., 2002; VanHouten et al., 2010; Kim et al., 2016). Finally, when we disrupted the *Casr* gene in mammary tumors in MMTV-Cre; CaSR^lox/lox^; MMTV-PyMT mice, tumor PTHrP mRNA levels were significantly reduced (Kim et al., 2016). Together, these data demonstrate that activation of the CaSR increases PTHrP production by breast cancer cells in rodents and in humans, both *in vitro* and *in vivo*.

**Figure 2:**
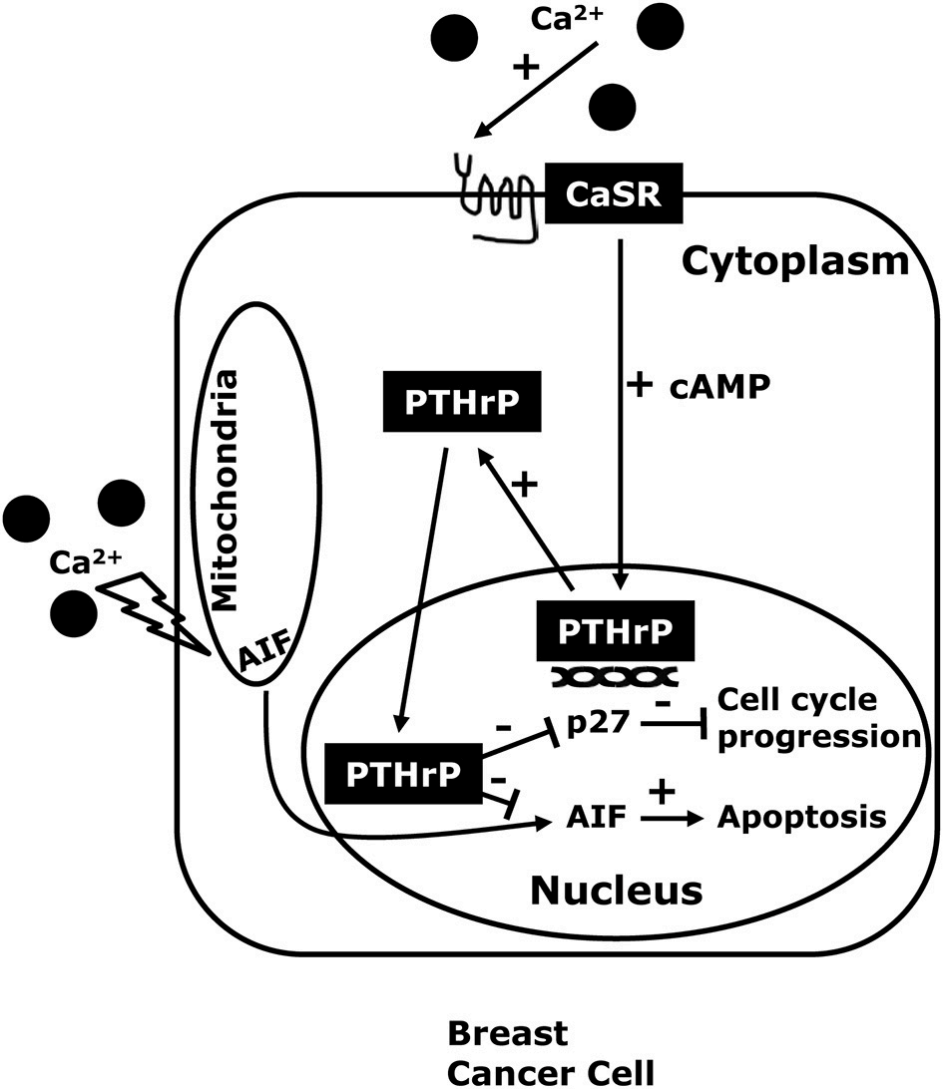
**The CaSR-nuclear PTHrP pathway**. CaSR activation in breast cancer cells, stimulates PTHrP production through increased intracellular cAMP levels and also promotes the proliferation and inhibits cell death in high extracellular calcium concentrations. The effects of the CaSR on tumor cell growth appear to be mediated by nuclear actions of PTHrP that decrease expression of the cell cycle inhibitor p27^kip1^ and that prevent nuclear accumulation of apoptosis-inducing factor (AIF) which activates apoptotic cell death.

**Figure 3:**
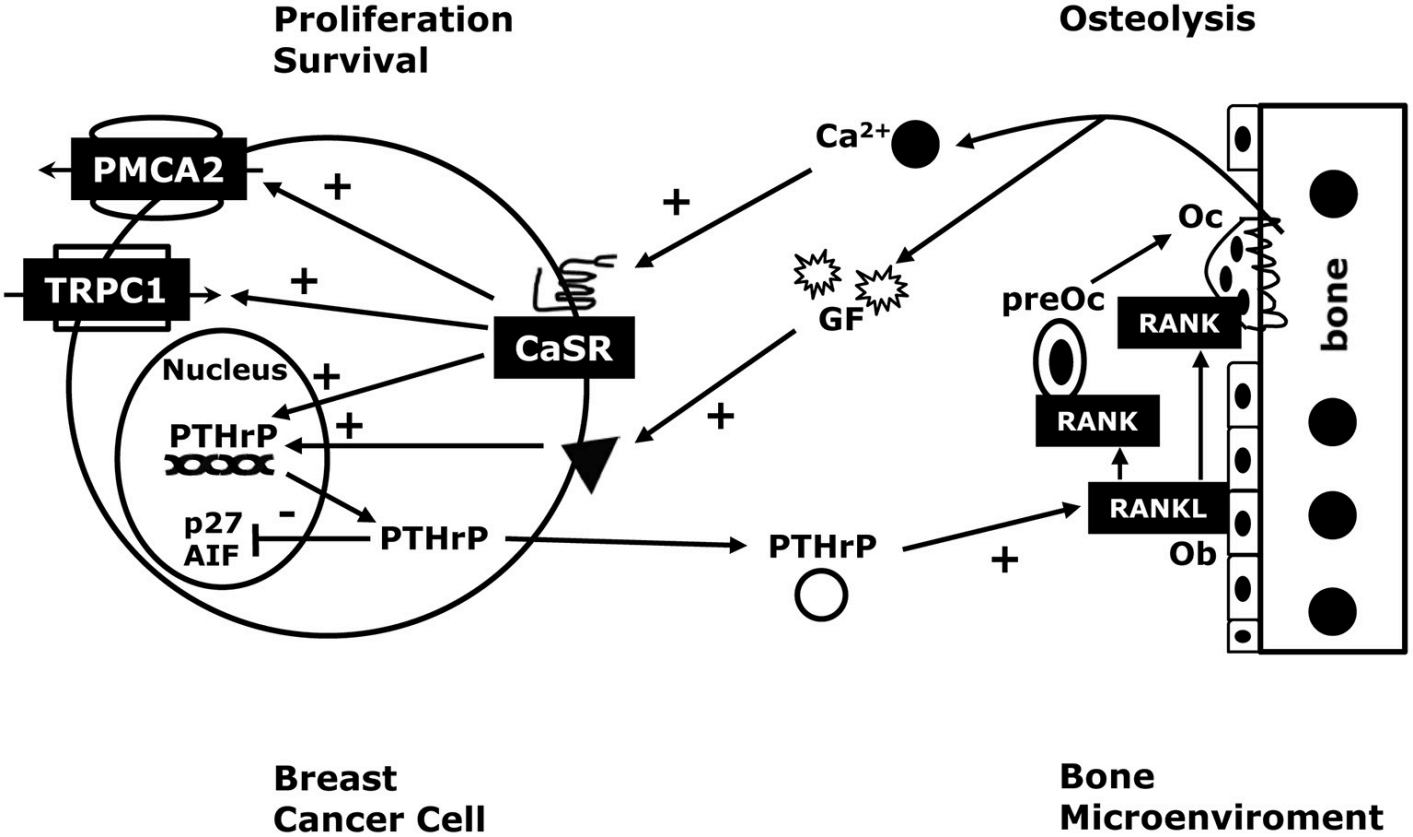
**The CaSR in breast cancer**. In breast cancer cells, stimulation of the CaSR increases PTHrP production. Therefore, the high local calcium concentrations surrounding bone metastases would be expected to stimulate PTHrP secretion, which would stimulate osteoblasts (Ob) to produce more RANKL, thereby driving more osteolysis and releasing growth factors (GF) from the bone matrix that stimulate tumor cell growth. As a result, activation of the CaSR facilitates a feed-forward, vicious cycle of bone resorption, tumor growth and osteolysis. In addition, the CaSR increase the PMCA2 calcium pump activity to protect the breast cancer cells from calcium-mediated apoptosis, and regulates the TRPC1 calcium channel, which can stimulate proliferation. Finally, as outlined in Figure 2, activation of the CaSR promotes breast cancer cell proliferation and enhances cell survival by activating a nuclear PTHrP signaling pathway that inhibits p27^kip1^ and AIF respectively. These findings suggest that extracellular calcium may promote tumor cell growth by activating the CaSR, which, in turn, can activate PTHrP-dependent and PTHrP-independent signaling pathways that stimulate cancer cell proliferation, inhibit cancer cell death and promote osteoclastic bone resorption.

Next, we found that knocking down either the CaSR or PTHrP in BT474 cells and MDA-MB-231 cells both inhibited proliferation and promoted cell death in response to high extracellular calcium levels. Stimulation of the CaSR promoted proliferation by reducing p27^kip1^ levels and increasing CDK2 activity while it promoted cell survival by inhibiting nuclear accumulation of apoptosis-inducing factor (AIF) (Figure 2). We also found that knocking out the CaSR inhibited the proliferation of mammary tumor cells in MMTV-Cre; CaSR^lox/lox^; MMTV-PyMT mice *in vivo*, slowing the growth of tumors. Furthermore, disrupting the CaSR inhibited growth and increased apoptosis in MMTV-PyMT tumor cells grown *ex vivo*, again by altering p27^kip1^ levels and nuclear AIF accumulation respectively. Interesting, although disruption of the PTHrP gene mirrored the effects of targeting the *Casr* gene, disruption of the Type 1 PTH/PTHrP receptor (PTH1R) did not (Kremer et al., 2011; Li et al., 2011; Kim et al., 2016). In addition, adding PTHrP to the media of cells could not rescue the loss of the CaSR, but transducing the same cells with a viral expression plasmid for wtPTHrP did rescue both the defects in proliferation and cell survival. By contrast, transducing the cells with a mutant form of PTHrP lacking the ability to translocate into the nucleus (ΔNLS-PTHrP) did not rescue the phenotytes caused by loss of the CaSR (Kim et al., 2016). In the aggregate, these findings clearly suggest that the CaSR affects breast cancer cell proliferation and apoptosis, at least in part, by stimulating the production of PTHrP, which, in turn, acts in the nucleus to regulate p27^kip1^ and AIF levels (see Figure 3).

### Does the CaSR contribute to bone metastases?

As discussed previously, Mihai et al. (2006) reported that the levels of tumor CaSR expression predicted the development of bone metastases. Larger clinical studies are needed to confirm these findings, but, in light of our recent findings, it is interesting to speculate on mechanisms by which the CaSR might promote the development of osteolytic bone metastases. In order to metastasize to the bone and grow in size, cancer cells must resorb the surrounding mineralized tissue, which requires them to stimulate osteoclast development and/or activity. Accelerated osteoclastic bone resorption, in turn, releases high levels of Ca^2+^ into the local microenvironment (Akhtari et al., 2008; Patel et al., 2011; Theriault and Theriault, 2012). For instance, some studies suggest that Ca^2+^ concentrations may increase to levels as high as 40 mM in the microenvironment surrounding actively resorbing osteoclasts (Silver et al., 1988; Berger et al., 2001). Therefore, it would be advantageous for a tumor cell to respond to Ca^2+^ in ways that would stimulate cell growth and further promote osteolysis. Given that PTHrP stimulates osteoclast differentiation and activity (Yin et al., 1999; Akhtari et al., 2008; Patel et al., 2011; Theriault and Theriault, 2012), increased PTHrP secretion in response to high Ca^2+^ levels would be expected to act in a paracrine fashion and contribute to accelerated osteolysis (Mamillapalli et al., 2008; Figure 3). Our findings also suggest that CaSR signaling can increase intracrine/nuclear signaling by PTHrP and directly stimulate tumor cell proliferation and enhance the ability of the cells to survive in the face of the elevated extracellular calcium concentrations resulting from active bone resorption (Figure 3). As discussed previously, increased bone resorption releases bone matrix-derived growth factors such as TGF-β, IGFs and FGFs, all of which can stimulate tumor cell growth and/or increase PTHrP production, defining a vicious cycle of osteolysis (Chirgwin and Guise, 2000; Mundy, 2002; Kremer et al., 2011). The majority of breast cancers that metastasize to bone are estrogen receptor (ER)-positive so it is also interesting that activation of the CaSR has been shown to increase ER transcriptional activity and enhance the effects of estradiol in MCF-7 cells (Journe et al., 2004; Leclercq, 2012). All of these observations demonstrate that CaSR signaling can exert proliferative, pro-survival and bone-resorbing activities that would be expected to enhance the progression of bone metastases (Figure 3). Therefore, although much research will be required to validate the above hypothesis, the CaSR-PTHrP axis may present new therapeutic opportunities to develop treatments for bone metastases from breast cancers.

## Conclusions

The CaSR has a central role in orchestrating systemic calcium homeostasis. However, it is also expressed in a variety of different organs such as the breast, where it may contribute to the regulation of cell proliferation, cell differentiation and cell migration in response to alterations in the extracellular environment. By regulating PTHrP production in the lactating mammary gland, the CaSR allows mammary epithelial cells to actively participate in reordering systemic calcium and bone metabolism to provide calcium for milk production. In a similar fashion, the CaSR appears to regulate PTHrP production by breast cancer cells, which causes osteolysis and stimulates the growth of tumor cells, contributing to the pathophysiology of osteolytic bone metastases. It is unlikely that PTHrP mediates all of the effects of the CaSR on breast cancers but, at this point, relatively little is known about the direct effects of CaSR signaling on breast cancer cell behavior, and many of the initial observation have been contradictory. Thus, more work will be required to fully elucidate the role of the CaSR in both normal and malignant breast cells and to determine whether targeting the CaSR-PTHrP axis would be an effective strategy against breast cancers.

## Author contributions

WK and JW both contributed intellectually to the development of this review, including drafting and revising the manuscript. Both approved the final version to be published.

### Conflict of interest statement

The authors declare that the research was conducted in the absence of any commercial or financial relationships that could be construed as a potential conflict of interest.
